# What Do We Know About Immune System Aging from Human and Animal Studies?

**DOI:** 10.3390/ijms27136037

**Published:** 2026-07-05

**Authors:** Marta Cąkała-Jakimowicz, Anna Domaszewska-Szostek, Monika Puzianowska-Kuźnicka

**Affiliations:** 1 Department of Human Epigenetics, Mossakowski Medical Research Institute, Polish Academy of Sciences, 02-106 Warsaw, Poland; mcjakimowicz@imdik.pan.pl (M.C.-J.); adomaszewska@imdik.pan.pl (A.D.-S.); 2 Department of Lifestyle Medicine and Longevity, School of Public Health, Centre of Postgraduate Medical Education, 01-826 Warsaw, Poland

**Keywords:** immunosenescence, inflammaging, aging

## Abstract

Aging is accompanied by complex structural and functional immune system changes driven by genomic instability, epigenetic alterations, mitochondrial dysfunction, telomere attrition, loss of proteostasis, deregulated nutrient sensing, and the accumulation of senescent cells exhibiting a senescence-associated secretory phenotype, which altogether lead to severe consequences including altered antimicrobial defense, the overproduction of autoantibodies, and chronic, low-grade inflammation (inflammaging). In this article, we summarize age-related alterations in the function of primary and secondary lymphoid organs, including the bone marrow, thymus, spleen, and lymph nodes. The involution of these organs leads to impaired hematopoiesis, reduced production of naïve lymphocytes, and immune microenvironment disruption. We also describe aging-related impairment of the activity of neutrophils, macrophages, dendritic cells and natural killer cells, as well as dysregulation of T and B lymphocyte responses. Specifically, these alterations include a decline in naïve cell populations, an accumulation of memory and exhausted cells, and a reduction in the diversity of antigen receptors. Consequently, older individuals exhibit increased susceptibility to infections, cancer, and autoimmune diseases, along with diminished vaccine efficacy. Understanding the mechanisms underlying immune aging could lay the foundation for developing therapeutic strategies and lifestyle interventions to mitigate the adverse effects of this unfavorable process.

## 1. Introduction

**Figure 1:**
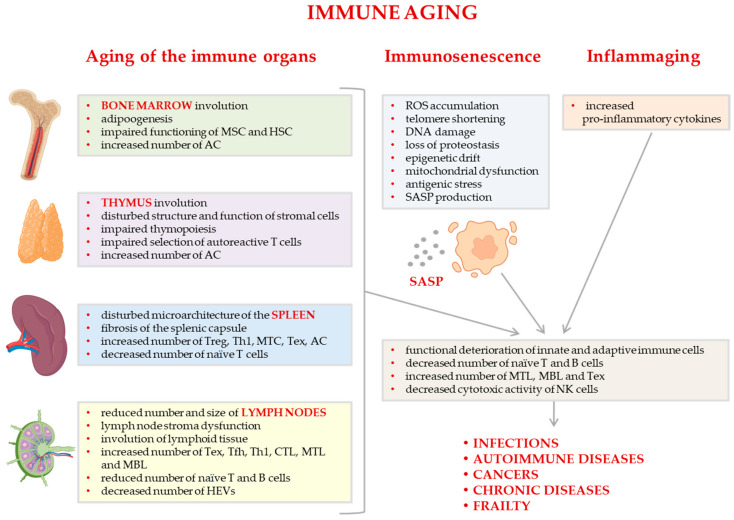
Age-related remodeling of the immune system and its consequences. AC: apoptotic cell; CTL: cytotoxic T lymphocyte; HEV: high endothelial venule; HSC: hematopoietic stem cell; MBL: memory B lymphocyte; MSC: mesenchymal stromal cell; MTL/MTC: memory T lymphocyte; NK: natural killer cell; ROS: reactive oxygen species; SASP: senescence-associated secretory phenotype; Tex: exhausted T lymphocyte; Tfh: follicular helper T lymphocyte; Th1: helper 1 T lymphocyte; Treg: T regulatory lymphocyte. Created in BioRender. Puzianowska-Kuznicka, M. (2026) https://BioRender.com/zn9ehjd.

Age-related involution of immune system organs (bone marrow, thymus, spleen, and lymph nodes) and lymphatic vessels as well as immune cell senescence are caused by numerous and diverse factors, with the dominant role played by the accumulation of DNA damage, epigenetic alterations, mitochondrial dysfunction, telomere attrition, loss of proteostasis, deregulated nutrient sensing, and other pathologies in cells of the innate and adaptive immune systems [1,2]. It manifests itself through a deterioration in the function of innate immune cells including neutrophils, dendritic cells (DCs), and macrophages; a reduction in the number of naïve T and B cells; attenuation of B and T lymphocyte function; and a reduction in the cytotoxic activity of natural killer (NK) cells. This leads to changes in the production of pro- and anti-inflammatory cytokines, with a relative predominance of pro-inflammatory ones and, consequently, to age-related, low-grade inflammation [3,4,5,6,7], as well as increased autoimmune processes with concomitant immunological deficits, which increase susceptibility to infectious diseases and the risk of infectious trauma complications, and also reduce responsiveness to vaccinations. In addition, the response to cancer cells is insufficient [8,9]. All these phenomena contribute to the complex age-related remodeling of the immune system. Immunosenescence, a component of immune aging, affects all immune system cell populations [10]; while some immune cells experience a decline in their function, other cell types adopt a hyperreactive, pro-inflammatory phenotype [11]. Senescent cells expressing markers specific to cellular senescence, such as increased activity of lysosomal β-galactosidase (SA-β-Gal), phosphorylated histone H2AX (γH2AX), and p53-binding protein 1 (53BP1), are characterized by altered morphology, cell cycle arrest, and apoptosis. Furthermore, they secrete the senescence-associated secretory phenotype (SASP), which includes pro-inflammatory cytokines and other factors that exacerbate age-related chronic inflammation [12]. In this article, we summarize data on the impact of aging on the immune system’s organs and cells, and their function, which contributes to the increased risk of age-related chronic diseases (Figure 1).

## 2. Methodology

This narrative review was based on results of PubMed/MEDLINE, Web of Science, and Scopus database searches, which covered publications from 1967 to 2026 and included studies examining age-related immune system changes in humans and vertebrates, with a focus on laboratory mice (*Mus musculus*) and rats (*Rattus norvegicus*). The following keywords were used: “immunosenescence,” “inflammaging,” and “immune aging,” in combination with terms referring to specific organs and cell populations discussed in the manuscript: “bone marrow aging,” “thymic involution,” “spleen aging,” “lymph node aging,” “neutrophil aging,” “macrophage aging,” “dendritic cell aging,” “NK cell aging,” “T cell aging,” and “B cell aging.” Original research articles and review articles published in English in peer-reviewed journals were included. Studies that included both clinical and population-based data from human studies and data from experimental animal models were included to identify common mechanisms and species-related differences.

## 3. Aging of the Immune Organs

The immune system is a complex network of organs, tissues, and cells that maintains the body’s integrity by removing foreign antigens and dead or dysfunctional cells [7,13]. It is estimated that 7% of the genes in the human genome are dedicated solely to its function and maintenance [14]. The bone marrow, thymus, and secondary lymphoid organs undergo aging [15,16]. Involution of lymphoid organs weakens interactions between stromal cells and lymphocytes, hindering the formation of naïve T cells, impairing the effective elimination of autoreactive lymphocytes, compromising the immune response to newly encountered foreign antigens, and weakening immunological memory. Consequently, the ability to fight infectious diseases and cancer cells, as well as to eliminate senescent cells, weakens [17,18,19,20,21].

### 3.1. Bone Marrow Aging

Bone marrow is a spongy tissue in the vertebral column, skull, and long bones, home to self-renewing pluripotent stem cells that give rise to the hematopoietic system [22]. Hematopoietic stem cells (HSCs) can differentiate into myeloid cells (granulocytes, monocytes, and dendritic cells) or lymphoid cells (T and B lymphocytes and NK cells) [23,24]. Mesenchymal stromal cells (MSCs) are one of the main components of the bone marrow microenvironment, promoting tissue homeostasis and balanced hematopoiesis, as well as modulating innate and adaptive immune responses [25]. Bone marrow involution occurs with aging, as demonstrated in detail in mouse models and humans. Aging HSCs exhibit reduced clonogenicity and self-renewal capacity, impaired function, and a predominance of the myeloid lineage over the lymphoid lineage, thereby reducing the potential to generate naïve T cells [26,27]. Age-related changes in MSCs [25] result in loss of differentiation potential, reduced proliferation [28], impaired autophagy, mitochondrial dysfunction, and SASP secretion [29,30,31]. Aging MSCs are less effective at inhibiting T cell proliferation, which is associated—among other things—with a decrease in the activity of the key enzyme that quells the immune response, indoleamine 2,3-dioxygenase (IDO) [32]. In a study in humans, MSCs from individuals aged 55 years or older were larger, more granular, and had altered morphology compared to MSCs from individuals aged 18 years old. Moreover, they expressed elevated levels of β-Gal, were characterized by SASP, accumulation of reactive oxygen species (ROS), and elevated levels of the cell cycle regulatory proteins p16 and p21, indicating that their ability to divide was inhibited. They showed impaired colony formation (increased population doubling time), reduced clonogenicity, and secreted increased amounts of IL-6 and IL-8 upon stimulation. MSCs from older donors lose their ability to inhibit M1 macrophages that present a pro-inflammatory phenotype, which may lead to the development of autoimmune and neoplastic diseases [33].

Increased adipogenesis [34] and an increased number of apoptotic cells [35] have been observed in aging human bone marrow. In aged mice, adipogenic differentiation of bone marrow stromal cells was accompanied by the induction of receptor activator of NF-κB ligand (RANKL) expression. Cells expressing the preadipocyte marker Pref-1^+^ were also RANKL^+^, and their number increased with age. Furthermore, this process was accompanied by a decrease in osteoprotegerin expression, and these cells were capable of generating osteoclasts from bone marrow macrophages. Therefore, the ability of preadipocytes to express RANKL and support osteoclastogenesis may partially account for the progression of bone marrow and bone destruction with aging [36]. Pref-1^+^RANKL^+^ cells produce several factors that influence hematopoiesis and increase production of myeloid lineage cells [7]. A significant increase in the number of Mac-1^+^ myeloid macrophages, which produced significantly less TNF-α than macrophages from young rodents, was demonstrated in aged mice, which indicates that the number of macrophages with an impaired ability to produce or release cytokines increases in the aging bone marrow, which may contribute to the age-related decline in hematopoietic reserves [37]. In humans, the ratio of NK cell precursors to T lymphocyte precursors increases with age. Remodeling of the NK cell subpopulation is manifested by a gradual decrease in the number of immature CD56^bright^ NK cells and an increase in the number of highly differentiated CD56^dim^CD57^+^ NK cells. Telomere loss and decreased telomerase activity may lead to decreased proliferation [38,39]. Changes in the aging bone marrow are exacerbated by the accumulation of senescent cells [40]. In mouse models, ROS produced by senescent cells have been shown to cause DNA breaks in hematopoietic stem cells. Deficiencies in DNA repair mechanisms further reduce the functional capacity of hematopoietic stem cells with aging. Defective stem cells cope relatively well under quiescent conditions but fail in stressful situations, as evidenced by insufficient proliferative potential, increased apoptosis, and eventual functional exhaustion [41].

### 3.2. Thymus Involution

Progenitor T cells from the bone marrow migrate to the thymus, where they undergo selection, transforming into naïve T cells [42]. Progenitor T lymphocytes have been identified in the human fetal thymus as early as 9 weeks of gestation, while mature T cells appear in the thymus at 12–13 weeks and in the spleen and lymph nodes at 24 weeks [43,44]. Human regulatory T cells (Treg) have been detected in the fetal thymus at 12 weeks and in lymph nodes at 14 weeks of gestation [45].

The fully developed thymus in humans and adult rodents consists of distinct regions that coordinate the stages of thymocyte differentiation. This process requires close interaction between thymocytes and the stromal microenvironment, which consists of thymic epithelial cells (TECs), endothelial cells, mesenchymal cells such as fibroblasts, dendritic cells, innate lymphoid cells, and macrophages. Thymocyte development and negative selection against autoreactive antigens are initiated in the thymus cortex and completed in the thymus medulla [46,47]. Medullary TECs (mTECs) play a key role in negative selection of thymocytes due to their ability to express a wide range of tissue-specific antigens (TSAs) and to mediate the elimination of autoreactive T lymphocytes. TSA expression by mTECs is controlled by epigenetic and transcription factors, the most well-known of which is the immune regulator (AIRE) [48,49,50]. AIRE expression ultimately selects for a diverse but self-tolerant T cell receptor repertoire [51].

The thymus reaches its maximum size approximately 1 year after birth, then decreases at a rate of 3% per year until middle age [52,53,54]. The mechanisms underlying thymic atrophy are not yet fully understood, although aging, infectious diseases, and malnutrition have been suggested to play a role [55]. Profound changes in the thymus can be observed in protein-, vitamin-, and trace element-deficient states, and especially in zinc deficiency [56]. Severe thymic atrophy with loss of cortical thymocytes is a consistent finding at autopsy in malnourished individuals [57,58]. Notably, thymic atrophy associated with malnutrition appears to be reversible if an appropriate diet is provided [59]. Similar atrophic changes in the thymus occur in acute infectious diseases, leading to apoptosis of immature CD4^+^CD8^+^ thymocytes [60].

Thymic progenitor cells are the primary target of the aging process, whereas the function of mature thymic epithelial cells is less impaired [61]. Palmer et al. demonstrated that the age-related decline in T lymphocyte production due to thymic involution is a major risk factor for many human cancers and infectious diseases [62]. Reduced naïve T lymphocyte production has been demonstrated in older mouse models. Due to their deficiency and abnormal maturation, the number of Tregs decreases, and autoreactive lymphocytes enter the circulation, increasing the risk of autoimmune diseases [54,63].

Kousa et al. described the appearance of two atypical states of thymic epithelial cells in aged mice, in which the cells formed high-density clusters of peri-medullary epithelium devoid of thymocytes. Furthermore, features of the epithelial–mesenchymal transition were observed, which were associated with decreased expression of the forkhead box protein N1 (*FOXN1*) gene, which is crucial for epithelial cell differentiation and function and important for thymic regeneration after injury [64].

With age, not only does the number of immunologically active thymic cells decrease, but the microenvironment of its stromal cells also becomes impaired. Corticomedullary junctions undergo atrophy, fibroblasts and fat cells expand, and the perivascular space increases [65,66]. In a study of adult human thymic tissues, epithelial cells stained positive for SA-β-Gal, and the tissue itself also stained strongly positive for γH2AX and 8-oxoguanine [67], indicating DNA damage and cellular senescence. Accumulation of senescent cells and their SASP enhances thymic involution. Administration of IL-6, a known SASP factor, has been shown to induce thymic atrophy with loss of cortical CD4^+^ and CD8^+^ thymocytes [68].

In male rats, a significant decrease in thymus weight is observed between 4 and 20 months of age, thus reducing the thymocyte population. Simultaneously, the relatively preserved sympathetic network leads to increased norepinephrine levels, in turn leading to disruption of the stromal microenvironment and inhibition of T cell differentiation, a key mechanism of immunosenescence [69]. Studies in male rats using genome-wide expression profiling at three age points (1, 4, and 18 months) have demonstrated that age-dependent transcriptional changes in the thymus are accompanied by global DNA hypomethylation, decreased histone H3K9 methylation, increased genomic instability, and increased apoptosis in 18-month-old animals compared with younger ones. These changes coincide with a significant disruption in the composition of the thymic resident T lymphocyte population, which directly impairs the generation of a pool of mature immunocompetent cells and constitutes a fundamental component of immunoaging. Furthermore, transcription factors closely associated with T lymphocyte homeostasis, activation, and differentiation, including MYC, are expressed at significantly lower levels in the thymus of aged rats, which is associated with the progressive loss of thymopoietic capacity [70]. These phenomena are consistent with a general model in which thymic involution drastically limits thymopoiesis through cell loss and microarchitectural damage, which, in turn, results in a reduced pool of naive T lymphocytes, narrowing of the TCR repertoire, and the accumulation of senescent immune cells, resulting in impaired adaptive immunity and increased susceptibility to infection, autoimmunity, and cancer [54].

### 3.3. Spleen Aging

The spleen is a secondary lymphoid organ where the immune response to bloodborne antigens is initiated. It consists of red pulp and white pulp surrounded by a fibrous capsule. The red pulp is separated from the white pulp by a marginal zone and is responsible for the removal of old and damaged platelets, erythrocytes, and apoptotic cells by splenic macrophages. The white pulp, in turn, consists of lymphoid tissue [71,72,73]. The spleen is responsible for both innate and adaptive immune responses [74]. Rodrigues et al. examined the structural arrangement of elastin fibers in the splenic capsule from 16 individuals aged 1 month to 76 years. In infants, elastin fibers were homogeneously interwoven with collagen fibers, which stabilizes the capsule during splenic growth and enlargement. With age, collagen fibers predominated on the outer surface of the capsule over elastic fibers, with the latter being more visible in the deep lamina of the splenic capsule. In older individuals, elastin fibers shortened, fragmented, and thickened. The progressive decline in elastin fibers in the splenic capsule with age may limit its distension and contribute to its involution [75]. In mice, spleen cellularity, microarchitecture, and cell localization change with age. Blurring of the boundaries of the compartments characteristic of T and B cells in the white pulp and changes in the organization and function of stromal cells, marginal zone macrophages, and metallophilic macrophages can be observed. Marginal zone macrophages no longer form a continuous border along the marginal zone [76].

Recruitment of CD4^+^ T lymphocytes to the aging spleen is impaired and coincides with reduced production of homeostatic chemokines such as CCL21 and CXCL13 by splenic stromal cells [18]. Aging splenic follicular dendritic cells are less dense and demonstrate an impaired ability to capture and retain immune complexes [76,77]. This contributes to impaired germinal center formation and antibody production. Germinal centers are small or absent [78,79,80]. Thinning of the capsule and a reduction in the number and size of B cell follicles have been observed in the spleens of people aged >70 years [81]. A reduced prevalence of spleen marginal zone B cells, which generate a rapid antibody response to T-cell-dependent and -independent antigens, is associated with a poor antibody response to bacterial capsular polysaccharides and an increased risk of infection with *Streptococcus pneumoniae*. This is important because pneumococci remain the most common cause of community-acquired pneumonia worldwide and one of the leading causes of infectious mortality in people over 65 years of age [82].

Flow cytometric evaluation of cell suspensions isolated from the spleens of old mice have shown a significant increase in the percentage of regulatory CD4^+^FOXP3^+^ T cells (Treg), exhausted CD45^+^CD3^+^CD4^+^PD-1^+^TIM3^+^ T cells (Tex), helper 1 CD45^+^CD3^+^CD4^+^CXCR3^+^ T cells (Th1), effector memory CD4^+^ and CD8^+^ T cells (TEM), central memory CD8^+^ T cells (TCM), and memory B cells, as well as a significant decrease in the percentage of naïve CD45^+^CD3^+^CD4^+^CD62L^+^CD44^−^ T cells, naïve CD45^+^CD3^+^CD8a^+^CD62L^+^CD44^−^ T cells, the CD4^+^ T cell subpopulation, and the CD4^+^/CD8^+^ T lymphocyte ratio compared to the spleens of young mice. The authors concluded that old mice experience chronic inflammation, constant antigenic stimulation, and reduced immune system functionality. Increased expression of the cyclin-dependent kinase inhibitors (CDKIs) p16^INK4a^ and p21^Waf1/Cip1^ has also been demonstrated in splenic CD45^+^ leukocytes, with significant increases in p21^Waf1/Cip1^ protein levels and increased ROS in splenic T and B cell populations [83].

Age-related accumulation of aging immunosuppressive cells and senescent cells and reductions in cytotoxic cell function all contribute to impaired immune surveillance. Park et al., using cultured mouse splenic stromal cells, demonstrated that IL-6 expression increases with age in both non-inflammatory and inflammatory states. These results suggest that splenic stromal cells may contribute to chronic inflammation during aging [84].

Changes in splenic immune cell populations have been analyzed in old rats (aged 18–24 months) compared to young rats (aged 3–5 months), and it has been demonstrated that aging leads to splenic atrophy, manifested by a significant decrease in organ weight and a reduction in the size of follicles. In old animals, an intensification of oxidative stress is observed, indicated by decreased activity of the total superoxide dismutase (T-SOD) enzyme alongside increased levels of malondialdehyde (MDA), a marker of lipid peroxidation in cell membranes. Within key structures of the spleen in older rodents, such as the periarteriolar lymphoid sheaths (PALS), marginal zone, and lymphoid follicles, there is a decline in the numbers of T and B lymphocytes, macrophages, granulocytes, IL-6, proliferating cells, and cells expressing TLR4. These negative changes are accompanied by an increase in the number of mast cells [85]. The degradation of functional lymphoid parenchyma described in rats directly mirrors the pathophysiological phenomena observed in the geriatric human population, where immunologically active spleen and lymph node tissue is progressively replaced by non-functional fibrous connective tissue. NK cell activity in the spleens of rats remains stable until 18 months of age, after which it declines rapidly and remains at a low level. The aging process induces profound regulatory changes, disrupting the interactions between NK cells, interferons, prostaglandins (especially PGE2), and macrophages. Removal of NK-suppressing macrophages which, instead of stimulating immunity, secrete inhibitory factors such as prostaglandin E2, allows for partial restoration of NK cell cytotoxic activity [86]. In parallel, T-cell-dependent immunity in the aging rat spleen undergoes profound suppression. The proliferative response to the mitogen phytohemagglutinin (PHA) and the production of IL-2 decline sharply in aging rodents, a phenomenon exacerbated by the extracellular suppressor activity of adherent splenic macrophages. In vitro co-culture assays have confirmed that adherent macrophages from old animals actively inhibit the proliferative capacity of young T cells, demonstrating that macrophages acquire immunosuppressive activity with age. In aging rats, this process is accompanied by a significant phenotypic shift toward memory T cells, manifested by a loss of OX-22/CD45RC antigen expression density on CD4^+^ helper T cells [87]. A longitudinal study of rats revealed the significant loss of sympathetic noradrenergic (NA) innervation in the spleen of older animals (21–27 months of age). Histochemical and immunocytochemical staining confirmed the progressive depletion of tyrosine hydroxylase (TH)-positive nerve fibers and decreased norepinephrine levels starting by 17 months of age. Parallel to the loss of innervation, a simultaneous decline in the density of OX19^+^ T lymphocytes and ED3^+^ macrophages was observed within splenic cellular compartments. This remarkably synchronous decline in both neural and immune elements provides evidence for dynamic interactions between the nervous and immune systems. These findings support a bidirectional neuroimmune interaction in the aging spleen, in which progressive immunosenescence and sympathetic denervation mutually reinforce each other [88].

### 3.4. Aging of Lymph Nodes

There are approximately 800 lymph nodes (LNs) in the human body. They play a key role in the innate and adaptive immune response. Their structure ensures efficient uptake, processing, and response to foreign antigens, thereby inducing a long-lasting adaptive immune response. The lymph node structure is composed of a cortex and a medulla surrounded by a connective tissue capsule, beneath which lies a macrophage-rich subcapsular sinus. Lymph containing immune cells and antigens reaches the node via afferent lymphatic vessels and is drained via efferent lymphatic vessels. Some cell subpopulations reach the nodes via the bloodstream, through high endothelial venules (HEV) [89]. The cortex of the node consists of an outer (cortex) and inner part (paracortex). The outer cortical layer, with its follicles, contains mainly CXCR5-expressing B cells and is rich in CXCL13, a chemokine that attracts B lymphocytes. In response to antigen exposure, B lymphocytes undergo intensive proliferation in the germinal centers of follicles, which are organized microstructures within B cell follicles where memory B cells and long-lived antibody-secreting plasma cells are generated, and where hypermutation and clonal expansion of B cells occur. These processes lead to the development of antigen-specific immunological memory [90]. The paracortex node is rich in the CCL19 and CCL21 chemokines, which act as chemotactic signals for T lymphocytes expressing the CCR7 receptor on their surface. Interactions with antigen-presenting dendritic cells activate T lymphocytes, which then proliferate to form clones of cells with high antigen specificity; in the paracortical zone, this process is crucial for initiating an effective immune response, both cellular and humoral [91].

Aging causes a reduction in the number and size of lymph nodes, as well as their structural disorganization [92]. Degenerative changes include the loss of lymphoid tissue in both the cortex and paracortex, a reduction in the number and size of germinal centers, and changes such as hyalinization, fibrosis, adipocyte deposition, and a decrease in the number of HEVs. Aging follicles are smaller, have irregular shapes, and shift toward the medulla, which may impair B-cell maturation and antibody production [93]. The percentage of B cells increases, while the percentage of T cells decreases with age [83]. In a study of superficial inguinal lymph nodes isolated from 41 deceased patients aged 17 to 98 years, a progressive reduction in the number of B and T lymphocytes with age was observed. In the lymph nodes of elderly individuals, an increase in connective tissue replacing the lymphoid tissue was observed [94]. Pan et al. studied 161 human head and neck lymph nodes and also described their progressive degeneration [95].

An analysis of mesenteric lymph nodes from rats ranging from 5 to 37 months of age revealed progressive and profound structural disorganization of the organ, with the most drastic tissue architecture alterations observed between 12 and 37 months of age. This process was characterized by a loss of cellularity in the cortical zone, a marked decrease in the number of germinal centers, and a pathological widening (distension) of the medullary sinuses caused by a loss of elasticity of the medullary sinus walls. Additionally, a reduction in the cortical area-to-medullary area ratio was noted, along with intensive infiltration of fibroblastic cells in both of these structures. These results clearly indicate that, in this species, aging leads to global disruption of the lymph node microenvironment, which may serve as a crucial extrinsic factor responsible for the decline in lymphocyte immune functions [96].

Similar to the spleen, a significant reduction in the percentage of naïve CD4^+^ and CD8^+^ T cells and naïve CD19^+^IgD^+^CD27^−^ B cells has been observed in the lymph nodes of aged mice. In addition, an increase in the percentage of exhausted CD45^+^CD3^+^CD4^+^PD^−^1^+^TIM3^+^ T cells (Tex), follicular helper CD45^+^CD3^+^CD4^+^CD154^+^CXCR5^+^ T cells (Tfh), helper type 1 CD45^+^CD3^+^CD4^+^CXCR3^+^ T cells (Th1), cytotoxic CD8^+^ and CD4^+^ T cells (CTL), central memory CD4^+^ and CD8^+^ T cells (TCM), effector memory CD4^+^ and CD8^+^ T cells (TEM), and memory CD19^+^IgD^−^CD27^+^ B cells was observed compared to young mice. The authors suggested that the increased percentage of memory cells in lymph node suspensions from aged mice may indicate that the aging organism is chronically exposed to various pathogens. In aged mice, high expression of p16^INK4a^ and p21^Waf1/Cip1^ was observed in CD45^+^ leukocytes in lymph nodes [83].

Stromal cells, which constitute the scaffold for immune cell migration, also change with age. An altered distribution of marginal reticular cells (MRCs) and a reduced network of follicular dendritic cells (FDCs) have been demonstrated in the lymph nodes of aged mice. This may affect immune complex uptake, hinder germinal center formation, and disrupt antigen presentation. Increased expression of the p21^Waf1/Cip1^ senescence marker in lymph node stromal cells of aged mice and increased immunofluorescence for ROS in fibroblastic reticular cells (FRCs) and lymphatic endothelial cells (LECs) of lymph nodes have also been observed [83]. Lymphatic endothelial cells (LECs) and HEVs present altered permeability, the accumulation of senescent cells, and increased inflammation, which may adversely affect the migration and recruitment of immune cells, such as naïve T cells [97]. Progression of lipomatosis leads to HEV remodeling, gradual loss of the lymphatic network in the lymph node medulla, and widespread dysfunction of the lymph node stroma [98].

A reduced ability of FDCs to capture and retain antigens has been demonstrated [99,100]. In addition, the production of IgM and IgG antibodies by B lymphocytes is reduced, and antibodies exhibit reduced affinity for antigens [101,102,103]. In 18–36-year-old and 65–75-year-old humans vaccinated against influenza, fivefold lower antibody production was demonstrated in the serum of older individuals. In a mouse model, fewer antigen-bearing dendritic cells were found in the draining lymph node of older animals after immunization, and those present had reduced expression of the CD80 and CD86 costimulatory proteins. The number of B lymphocytes in germinal centers was tenfold lower in old mice ten days after immunization compared to younger adult mice [104].

Sonar et al. demonstrated that secondary lymphoid organs in mice age asynchronously. Early atrophy of skin draining lymph nodes, which begins as early as 6 to 9 months of age, significantly precedes the involution of the spleen and deep tissue draining lymph nodes, which occurs later, at 18 to 20 months. Key mechanisms of this process include the degradation of the fibroblastic reticular cell (FRC) network and loss of the lymph nodes’ capacity to retain naïve T lymphocytes. Consequently, massive translocation of these cells to the peripheral blood occurs, accompanied by a phenotypic shift towards CCR7^lo^ S1P1^hi^ (low lymph node homing receptor CCR7 and high sphingosine-1-phosphate receptor S1P1), favoring egress into the peripheral blood). These phenomena result in an early decline in local immunity and an impaired de novo immune response to intradermal vaccination, detectable in mice from as early as 7 to 8 months of age [105]. Furthermore, Sonar et al. also demonstrated that age-related oxidative stress and mitochondrial dysfunction in lymph node stromal cells critically impairs the homeostatic maintenance and function of peripheral T lymphocytes in mice. All three major stromal cell subsets—FRCs, LECs, and blood endothelial cells (BECs)—were found to exhibit elevated levels of mitochondrial ROS (mROS). This was accompanied by a reduced mitochondrial membrane potential and an increased total mitochondrial mass in older mice. Moreover, aged FRCs produced elevated levels of cytoplasmic ROS. These intracellular metabolic defects directly limit the capacity of old lymph node stromal cells to support the survival of naive T cells in vitro. Ex vivo experiments have demonstrated that treating old stromal cells with antioxidant N-acetylcysteine (NAC), the mROS-reducing compound mitoquinone, or the mitophagy-inducing urolithin A effectively alleviated this homeostatic support defect. Crucially, in vivo antioxidant treatment of aged mice with NAC restored the numbers of antigen-specific CD8^+^ effector T lymphocytes to younger adult levels. Furthermore, this treatment successfully rescued the ability of T cells to produce granzyme B in response to antigenic challenge, highlighting potential therapeutic strategies to reverse immunosenescence [106].

An analysis of sex-related differences in immunosenescence in rats demonstrated that alterations in the lymph node T cell compartment progresses faster in males, as confirmed by a higher summary aging index. With increasing age, both sexes exhibited a decrease in total lymph node T cell counts and a shift in the CD4^+^/CD8^+^ ratio towards CD8^+^ T cells, alongside a shift from a naive phenotype (which includes recent thymic emigrants and mature naive cells) toward a memory/activated T cell phenotype. Notably, all these alterations were more pronounced in male rats. Furthermore, the frequency of regulatory CD25^+^Foxp3^+^ cells increased among lymph node CD4^+^/CD8^+^ T cells with aging, reflecting, at least partly, the enhanced conversion of effector T cells into regulatory cells, which was also more prominent in male rats. Higher levels of oxidative damage and pro-inflammatory cytokine expression in the lymph nodes of aged males likely contributed to a greater accumulation of pro-inflammatory, senescent CD28^−^ cells expressing CD11b, as well as exhausted PD-1^high^ T cells (particularly within the CD8^+^ subset), compared to age-matched females. Finally, the study demonstrated that these indices of lymph node T cell aging strongly correlated with those observed in peripheral blood [107].

In summary, these findings in humans and rodent models demonstrate that lymph node involution is a complex, asynchronous process wherein tissue architecture degradation, metabolic mitochondrial dysfunction of stromal cells, and sex-specific variances concurrently drive the early decline of peripheral T cell homeostasis and functional immunity.

## 4. Immune Cells Senescence

### 4.1. Innate Immune System Cells

Innate immune cells constitute the first line of defense against infections. Their role is to initiate the inflammatory response, phagocytose and remove pathogens, recruit NK cells, and support the maturation and migration of dendritic cells, which initiate the adaptive immune response. These cells cooperate with adaptive immune cells to generate an effective humoral response. With aging, changes in the expression and function of toll-like receptors (TLRs) and in signal transduction in innate immune cells have been observed, leading to their abnormal activation, reduced chemotaxis, phagocytosis, and intracellular killing of pathogens [108].

In the peripheral blood of elderly humans, a 48% depletion in the NK CD56^bright^ cell subpopulation was observed, leading to reduced production of regulatory cytokines and chemokines, impairing signaling and the recruitment of other immune cells [109]. The expanded NK CD56^dim^CD57+ cell subpopulation demonstrates high cytolytic capacity but reduced responsiveness to IL-12 and IL-18 signaling [110]. Furthermore, reduced expression of the cytotoxicity receptor NKp30 was observed in older individuals, along with decreased cytotoxic effector function in NK cells. NKp30 also plays a key role in regulatory signaling with dendritic cells; therefore, its deficiency may impair the development of adaptive immune responses [111].

In aging mice, changes in the NK cell population have been documented that share similarities with, but also exhibit mechanistic differences from, those observed in humans [112,113]. A multi-organ, high-parametric single-cell cytometric analysis across 12 tissues in young and aged mice revealed a consistent decline in NK cell numbers, as well as a decrease in the frequency of plasmacytoid dendritic cells in multiple peripheral organs, including the spleen and lymph nodes [113]. In the bone marrow of aged mice, the maturation process of NK cells was impaired due to defective stimulatory signals sent by bone marrow stromal cells. This resulted in features of NK cell immaturity in peripheral tissues, such as reduced in vivo proliferation, altered expression of activating and inhibitory receptors, and decreased levels of collagen-binding integrins. Although treating mice with IL-15/IL-15Rα complexes induced a massive numeric expansion of NK cells, most of these cells remained immature and failed to provide protection against mousepox virus infection [112]. This demonstrates that numeric cellular expansion alone is insufficient to restore NK cell function in aging organisms.

Dendritic cells present antigens and tumor cell fragments, serving as a major bridge between the innate and adaptive immune responses. They provide activation signals to T lymphocytes, which are essential for generating both cellular and humoral responses [114]. In older people, dendritic cells exhibit reduced capacity for antigen capture, phagocytosis, chemotaxis, migration, and cytokine secretion [115]. Sridharan et al. observed a weakened response of plasmacytoid dendritic cells (PDCs) isolated from the peripheral blood of older (65–90 years) healthy donors to influenza virus stimulation, resulting in impaired type I interferon (IFN-I) secretion. They also demonstrated a significant reduction in IFN-III production, which plays an important role in antiviral defense on mucosal surfaces, including those of the respiratory tract. With aging, PDCs exhibit impaired phosphorylation of the transcription factor interferon regulatory factor-7 (IRF-7) and a reduced ability to induce perforin and granzyme in CD8^+^ T lymphocytes, to secrete IFN-γ, and to induce proliferation of CD4^+^ and CD8^+^ T lymphocytes compared to cells from young individuals [116]. Myeloid dendritic cells (MDCs) from older individuals exhibit a reduced ability to phagocytose antigens through macropinocytosis and endocytosis, an impaired ability to migrate in vitro in response to the chemokines macrophage inflammatory protein-3 beta (MIP-3ß) and stromal cell-derived factor-1 (SDF-1), and significantly increased secretion of TNF-α and IL-6 induced by lipopolysaccharide (LPS). Reduced activation of the phosphoinositide 3-kinase (PI3K) pathway, which coregulates phagocytosis, cell migration, and TLR signaling in MDCs, may explain the dysfunction of innate immunity in older adults [117]. Furthermore, Agrawal et al. demonstrated the increased reactivity of dendritic cells from elderly individuals to self-antigen (human DNA), which in turn may contribute to chronic age-related inflammation [118]. Reduction in FcγRII receptors on FDCs and the development of a defective FDC network that retains few immune complexes impairs B cell proliferation, germinal center formation in lymph nodes, and antibody production, negatively impacting the development of an adaptive immune response [99,100,119].

A reduced number of Langerhans cells (LCs) in the epidermis of older individuals and their reduced migration induced by TNF-α have been demonstrated. As these cells play a key role in the skin’s immune response, age-related changes may impair skin immune function [120]. Specifically, aging mice showed impaired maturation and reduced numbers and densities of LCs. Although langerin expression and phagocytic capacity in aged LCs were increased compared to cells from young mice and their migration to draining lymph nodes was comparable, their total number in draining lymph nodes was significantly lower in older mice, which is most likely a result of their reduced numbers in the epidermis from which they migrated. Aging LCs showed an impaired ability to induce the proliferation of antigen-specific CD4^+^ and CD8^+^ T cells, and the miRNA expression profile in aged LCs differed compared to that of young LCs. During aging, miRNAs, by influencing TGF-β-dependent and -independent signaling pathways, contribute to developmental and functional changes in LCs, which may impair immune surveillance in the skin and contribute to age-related skin diseases such as cancer, allergies, infections, and autoimmune diseases [121]. 

Through single-cell RNA sequencing analysis of lymph nodes in young and aged mice after vaccination, researchers identified DC migration defects as a key cause of diminished immune responses in aging. These defects are accompanied by a specific gene expression profile associated with impaired CCR7-dependent DC migration. Studies have demonstrated that oral delivery of yeast-derived β-glucan-containing nanoparticles elevated chemokine receptor CCR7 expression in intestinal DCs. This increased receptor expression facilitated the trafficking of DCs to lymph nodes in response to CCR7 ligand (CCL19/CCL21) signals generated after immunization. Consequently, this targeted therapy effectively enhanced vaccine-induced immunity in aged animals. These findings reveal a fundamental mechanism of immune system aging and offer a noninvasive strategy to improve DC function and vaccine efficacy in seniors [122]. Aged murine DCs demonstrate impaired antigen cross-presentation and weakened CD8^+^ T cell priming compared to their younger counterparts. This dysfunction results from a reduced abundance of key DC subpopulations, diminished phagocytic capacity, and mitochondrial disorders, including increased ROS production and a drop in membrane potential. Reducing ROS levels in vitro partially restores cross-presentation capacity in aged DCs. Additionally, studies have shown that the age-associated decline in ATP content directly inhibits the process of antigen particle engulfment by dendritic cells. These findings suggest that immune system aging is not totally irreversible because it can be modulated through targeted metabolic interventions [123]. Both elderly humans and aged mice exhibit a profound impairment in Tfh cell differentiation following vaccination. This immunological deficit is directly preceded by poor activation of conventional type 2 dendritic cells (cDC2s), a defect rooted in diminished type I interferon signaling that prevents these cells from effectively supporting humoral immunity. Crucially, targeted treatment with a TLR7 receptor agonist fully restored cDC2 activation and stimulated proper germinal center formation in the lymph nodes of aged subjects. These findings unequivocally demonstrate that aging-associated defects in cDC2 and Tfh cell responses are not irreversible consequences of structural tissue deterioration, but, rather, can be pharmacologically corrected to enhance vaccine efficacy in the elderly population [104]. Grolleau-Julius et al. demonstrated that the reduced efficacy of DCs from aged mice against melanoma resulted from their impaired function rather than antigen presentation itself. Aged DCs exhibited a decreased capacity to stimulate T cells in vitro and a distinct in vivo migration defect linked to the CCR7-CCL21 axis. Additionally, their phenotypic analysis revealed a selective decrease in DC-specific/intracellular adhesion molecule type-3-grabbing nonintegrin (DC-SIGN) levels in aged cells. The adoptive transfer of aged DCs into young recipients confirmed weaker activation and reduced influx of CD8^+^ T cells into the tumor. Although increasing the number of transferred aged DCs partially restored their migration to lymph nodes, it did not change their intrinsic defect in T cell activation. The authors pointed to impaired T cell stimulation as the primary cause of reduced immunotherapeutic efficacy in aging subjects [124]. Aging has been found to impair antiviral immunity in mice by reducing IFN-α production by plasmacytoid dendritic cells (pDCs), resulting in defective clearance of the herpes simplex virus type 2 (HSV-2). The primary molecular cause is the defective upregulation of interferon regulatory factor 7 (IRF-7)—a key adaptor in the type I IFN pathway—during TLR9 activation, alongside elevated cellular oxidative stress. Mitigating oxidative stress partially reversed the defect and restored proper IFN-α production [125].

Neutrophils are the first cells recruited to sites of pathogen aggression, and thanks to their surface receptors, they are activated by a wide range of compounds that initiate their chemotaxis, phagocytosis, and ROS production. Upon stimulation, neutrophils produce lipids and immune mediators and can present antigens in the context of major histocompatibility complex (MHC) class I molecules [126]. Age-related chronic inflammation leads to epigenetic changes in neutrophils, contributing to abnormalities in their function, which, in turn, result in reduced phagocytosis [127], abnormal adhesion and chemotaxis [128], reduced release of neutrophil extracellular traps (NETs) [129], and TLR dysfunction [130]. Neutrophils are short-lived and undergo spontaneous apoptosis. Their lifespan and functional activity can be extended in vitro by granulocyte–macrophage colony-stimulating factor (GM-CSF) through Jak/STAT pathway activation. However, the protective effect of GM-CSF is not observed in neutrophils from elderly individuals. The increased susceptibility to apoptotic signaling with age may lead to premature neutrophil elimination, thereby weakening initial responses to infections or vaccinations [131]. Age-related changes in neutrophil function increase the risk of overall morbidity and mortality in aging populations [132].

Functional studies in aged mice have confirmed impaired phagocytosis of opsonized *Streptococcus pneumoniae* and diminished intracellular bacterial killing, the latter linked to reduced production of cathelicidin-related antimicrobial peptide (CRAMP), the murine equivalent of the human antimicrobial peptide LL-37. Additionally, the speed and directionality of chemotaxis are blunted in aged murine neutrophils, NETosis is impaired, and wound healing is defective in association with reduced expression of intercellular adhesion molecule 1 (ICAM-1), which is required for neutrophil extravasation and recruitment to sites of injury. Crucially, adoptive transfer of neutrophils from young mice into aged recipients restores resistance to pneumococcal infection, confirming that these functional deficits are neutrophil-intrinsic rather than driven solely by the aged systemic environment. To understand the underlying molecular mechanisms of these cellular deficits, recent comparative studies have mapped the internal communication networks of both species, revealing distinct, species-specific signaling disruptions. In old mice, for instance, such functional deficits are strongly dependent on the TLR4/LPS-priming pathway and are associated with diminished phosphatidylinositol (3,4,5)-trisphosphate (PIP3) production and downregulation of the adaptor protein myeloid differentiation primary response 88 (MyD88). In contrast, the molecular basis for the impaired apoptosis rescue observed in elderly humans—as introduced above—is specifically tied to the distinct dysfunction of the GM-CSF/(Jak/STAT) pathway [133].

Monocytes and macrophages are phagocytic cells crucial to the development of innate immunity that are also subject to the aging process; specifically, macrophages exhibit a decline in phagocytic function with age [134,135]. Reduced production of IL-6 and TNF-α has been demonstrated in human monocytes following TLR1/TLR2 stimulation [136,137], and a similar phenomenon has been observed in macrophages from aged mice in response to TLR4 signaling [138]. Furthermore, studies on human monocytes and murine macrophages have shown that HLA and MHC class II protein levels decrease with age [139,140]. Macrophages become chronically activated and adopt an inflammatory phenotype. Their abnormal function is associated with immune decline and diseases such as atherosclerosis, diabetes, fibrosis, immunosuppression, autoimmune diseases, cancer, and aging-associated inflammation [3,141]. Moss et al. demonstrated significant reductions in phagocytosis, migration, and chemotaxis in human monocyte-derived macrophages (MDM) from older donors (>50 years) compared to those from younger donors (18–30 years). These features were associated with decreased expression of the avian myelocytomatosis viral oncogene homolog transcription factor (c-MYC) and the upstream transcription factor (USF-1) in both humans and mice. This observation makes the MYC-USF1 axis a promising target for future immune rejuvenation therapies [142]. Aged macrophages demonstrate decreased surface expression of the M2 marker CD206 (mannose receptor) and upregulated expression of inducible nitric oxide synthase (iNOS), a hallmark of classically activated, pro-inflammatory M1 macrophages [143]. This shift is driven by the accumulation of oxidative damage and ROS, which activate NF-κB signaling and tip the balance toward M1-promoting transcription programs. Furthermore, SASP factors secreted by neighboring senescent cells act in a paracrine manner to polarize macrophages toward the M1 phenotype while simultaneously suppressing reparative M2 polarization by inhibiting the IL-4/IL-13/STAT6 signaling pathway [144]. Additionally, impaired efferocytosis driven by diminished receptor tyrosine kinase MerTK signaling and reduced autophagy in aged macrophages allows apoptotic debris to accumulate and undergo secondary necrosis. This process leads to the release of damage-associated molecular patterns (DAMPs) that amplify the inflammatory cascade [145]. A distinct population of senescent macrophages has been identified and characterized as a major source of sterile inflammaging. These cells exhibit high p21 (CDKN1A) expression and permanent cell cycle arrest induced by DNA damage or metabolic stress. Furthermore, they are characterized by elevated SASP secretion (including IL-6, IL-1β, MCP-1, MMP-9, and TNF-α), mitochondrial dysfunction, and sustained interferon signaling. Senescent p21^high^ macrophages accumulate specifically in the livers of aged mice and are also present in human cirrhotic liver [146]. Smith et al. developed a novel in vitro model of murine peritoneal macrophage senescence. Their study confirmed that macrophages cultured for 7 to 14 days acquire a senescence-like phenotype, characterized by decreased proliferation, accumulation of the cyclin-dependent kinase inhibitors p16^INK4A^ and p21^CIP1^, and enhanced SASP secretion. These changes were accompanied by a sharp decline in phagocytic capacity and the induction of glycolytic activity. In this model, chronic treatment with CB3, an anti-inflammatory thioredoxin-1 mimetic peptide, completely prevented the upregulation of p21^CIP1^ expression and allowed the cells to retain their proliferative activity on day 14. Implementing such innovative therapeutic strategies, alongside the targeted clearance of senescent macrophages using conventional senolytics, effectively reduced hepatic steatosis and chronic inflammation in preclinical models of metabolic diseases [146,147].

### 4.2. Adaptive Immune System Cells

The main cells of the adaptive immune system are T and B lymphocytes, which are responsible for the antigen-specific immune response. T cells recognize and destroy infected cells, while B cells produce neutralizing antibodies. CD4^+^ T lymphocytes are essential for coordinating humoral immunity, while cytotoxic CD8^+^ T cells directly participate in the cytotoxic response. Defects in the responses of both subpopulations have been observed during aging. Attenuated recognition of new antigens by T cells due to reduced TCR variability appears to be crucial. Furthermore, senescent cells begin to exhibit SASP features [2,148]. Age-related changes in the thymus result in the decreased production of naïve T cells and increased proportions and numbers of senescent and memory T cells [6]. Highly differentiated T cells that have experienced antigen exposure accumulate. These cells exhibit features similar to those of senescent cells, including shorter telomeres, accumulated DNA damage, and metabolic changes. During differentiation, both CD8^+^ and CD4^+^ T cells express CD57. It has been suggested that T cells first lose CD28 and then, in a second step, express CD57, thus generating a population of CD28^−^CD57^+^ T cells. Loss of CD28 expression is one of the hallmarks of T lymphocytes after repeated antigenic stimulation, but CD28^−^ T lymphocytes cannot be considered truly senescent because they are still capable of proliferation after appropriate stimulation [149].

Effector memory CD8^+^CD27^−^ T cells that re-express CD45RA (TEMRA CD8^+^ T cells) are a component of the aging immune system. Morphologically, they resemble naïve cells by expressing the CD45RA marker, but are functionally distinct. They exhibit high cytotoxicity and pro-inflammatory activity. They are characterized by features of cellular senescence, including the presence of CD57 and/or KLRG1 markers, phosphorylation of γH2AX, and SASP. They also exhibit mitochondrial dysfunction and reduced ATP levels, which result from autophagy inhibition via the p38 MAPK pathway. Despite their defective replicative potential compared to other memory T cells, they are potent producers of inflammatory cytokines (TNF-α, IFN-γ), which may fuel chronic inflammation [150]. Their accumulation is closely linked to chronic infections, especially CMV, which forces continuous lymphocyte activation [151,152]. In a study of supercentenarians (110 years old), T cell counts in this population were similar to those of control subjects aged 50 to 80, but their profiles were altered. However, in addition to the reduction in naïve T cells, CD4^+^ T cells, which primarily serve a helper function by coordinating the immune response and activating humoral mechanisms in cooperation with B lymphocytes, exhibited cytotoxic features. According to the authors, the transformation of helper CD4^+^ T cells to a cytotoxic phenotype can be viewed as an adaptation to the late stage of aging [153].

Mouse models have been indispensable for defining the mechanistic underpinnings of T cell aging. Using unified mathematical modeling of naive T cell dynamics from birth to old age in mice, Rane et al. analyzed how naive CD4^+^ and CD8^+^ T cell populations are maintained. Murine organisms do not rely on continuous proliferation to maintain a constant cell number. Instead, as the thymus undergoes age-associated involution, existing T cells progressively extend their lifespan. This extended survival partially offsets the decline in new thymic output. Researchers have observed that this dynamic differs slightly between lineages. Specifically, CD4^+^ helper T cells age, survive longer, and divide rarely throughout an organism’s life. In contrast, young CD8^+^ T cells disappear more rapidly during the first weeks of life. This initial loss has been linked to their direct recruitment into the immunological memory pool. Consequently, even modest perturbations in thymic output, such as those induced by infection, malnutrition, or corticosteroids, can have disproportionate long-term consequences for the size and diversity of the naive T cell pool in aging, a principle also applicable to humans [154]. With age, mice experience a drastic decline in TCR diversity. Due to continuous exposure to microbes and chronic low-grade inflammation, certain cell populations begin to dominate over others. In the spleen of aged mice, CD4^+^ lymphocytes lose their diversity because individual, random cells rapidly expand without any apparent foreign antigenic stimulus. However, this process does not occur uniformly throughout the body. While the spleen loses its ability to recognize new threats, the bone marrow of aged animals still maintains high immune cell diversity. This demonstrates that the immune system ages differently depending on the organ, and the bone marrow serves as a safe environment for long-lived memory T cells [155]. In TCR*δ*^CreER^ R26^ZsGreen^ strain mice, profound changes have been demonstrated in the gene expression profile of aging CD4^+^ and CD8^+^ T lymphocytes. Specifically, in both naive CD4^+^ and CD8^+^ lymphocytes, the aging process led to a significant impairment of cellular organelle functions (such as mitochondrial respiration and membrane envelope integrity). Conversely, a distinct increase in the activity of genes associated with lymphocyte activation and function has been recorded in aging memory cells. These changes are accompanied by elevated expression of immunosuppressive markers and immune checkpoints, including programmed cell death protein 1 (PD-1)**,** lymphocyte activation gene 3 (LAG-3)**,** and T cell immunoglobulin and mucin-domain containing-3 (TIM-3)**,** which clearly indicate T cell exhaustion. Furthermore, age significantly modifies survival mechanisms and cell death signals. While aging CD4^+^ memory lymphocytes exhibit a pro-apoptotic signature (promoting cell death), CD8^+^ memory cells activate anti-apoptotic genes (protecting against cell death). This asymmetric apoptotic regulation between CD4^+^ and CD8^+^ memory T cell fractions may underlie the characteristic skewing of the CD4/CD8 ratio observed in aged immune systems in both mice and humans [156].

Mononuclear cells isolated from the peripheral blood of healthy individuals demonstrate an age-correlated increase in SA-β-Gal activity. The largest increase has been observed in CD8^+^ T lymphocytes, with 60-year-olds showing an average of 64% of cells with high enzyme activity. These cells are characterized by dysfunctional telomeres, p16-mediated senescence, and impaired proliferation capacity [157]. To assess changes that precede functional decline with aging, Gong et al. used RNA-seq to analyze the transcriptomes of peripheral blood leukocytes from individuals of varying ages. The authors observed that the greatest age-related transcriptomic changes occurred in the early stage of T lymphocyte differentiation, i.e., naïve T lymphocytes, and, to a lesser extent, in central memory T lymphocytes and effector memory T lymphocytes. This study also provides a unified model of immune aging, in which the reprogramming of CD4^+^ and CD8^+^ T lymphocytes toward a Th2 phenotype with age promotes the reduced activation of effector B lymphocytes. By examining responses to influenza vaccination, the authors demonstrated that the effector memory CD27^−^ B lymphocyte subpopulation was characterized by reduced ROS signaling; lower expression of the lineage-defining genes *FCRL5*, *CD19*, and *MS4A1*; activation of the genes *ZEB2*, *TBX21*, and *BATF*; and altered expression of the immunoglobulin constant heavy G chain (*IGHG*) gene, which is associated with increased production of IgG2 relative to IgG3 (which is responsible for the early antiviral response) in the influenza-specific IgG antibody repertoire, explaining the reduced response to influenza vaccination in older individuals. The number of effector memory IgG^+^CD27^−^ B lymphocytes in older individuals following vaccination was also lower than in younger individuals. Impairment of B lymphocyte function due to age-related changes in T lymphocytes may contribute not only to a weakened response to vaccination but also to impaired immune tolerance and an increased risk of some autoimmune diseases [158].

Immunosenescence also affects B lymphocytes [159]. In humans, the number of naïve B cells decreases with age, while the number of memory B cells increases, weakening the ability to respond to newly encountered antigens [101]. Reduced B cell production may be related to reduced numbers and function of progenitor cells. In mice, the ability of B lymphocytes to proliferate in the presence of bone marrow stromal cells decreased after 24 months of age, as did the ability of stromal cells to support this proliferation. Furthermore, stromal cells have been observed to produce less IL-7, a key growth factor for B lymphocytes, with age [160,161,162], and aging mouse B lymphocyte precursors demonstrate limited expansion in response to IL-7 [163]. Although the best-known function of B lymphocytes is antibody production, they are highly effective antigen-presenting cells and are essential for the development of memory T cells [164]. IL-10-secreting B lymphocytes can prevent inappropriate immune stimulation leading to autoimmune diseases and limit the aggressiveness of immune responses [165]. Loss of diversity in the B lymphocyte repertoire has serious consequences for the integrity of the humoral immune system. In older people, the production of specific antibodies decreases, while that of nonspecific antibodies increases. In peripheral blood B lymphocytes from individuals aged 86–94 years, a decline in the diversity of B cell receptors (BCRs) recognizing specific antigens was demonstrated. This decline in B lymphocyte diversity was strongly correlated with overall health and frailty. A correlation was also observed with lower survival and vitamin B_12_ deficiency. The loss of diversity has been attributed to the proliferation of certain B cell clones, which may result from impaired production of new B cells in the bone marrow or the accumulation of cells that have already been exposed to antigens [101]. B-1 cells from healthy individuals over 65 years of age have shown significantly lower expression of the transcription factors XBP-1 and BLIMP-1, which are key regulators of B lymphocyte differentiation into plasma cells and of maintenance of proper function, compared to those from young individuals. Furthermore, higher levels of PAX-5 have been observed, which is characteristic of non-antibody-secreting B lymphocytes [166].

A comparative overview of aging-related changes across primary and secondary lymphoid organs and immune cell populations—encompassing evidence from human and animal models—and their shared molecular mechanisms, is presented in Table 1.

## 5. Clinical and Translational Implications

The structural and functional changes in immune organs and cells described in the preceding sections have direct consequences for health outcomes in older individuals. Age-related declines in naïve T and B cell output, reduced germinal center activity in lymph nodes, impaired dendritic cell migration, and the progressive accumulation of senescent cells collectively underlie increased susceptibility to infections, diminished vaccine efficacy, and higher risks of cancer, autoimmune diseases, and chronic inflammatory conditions. This section outlines the main translational implications of these findings and discusses therapeutic and lifestyle strategies that have shown promise in modifying the trajectory of immune aging.

### 5.1. Clinically Relevant Biomarkers of Immune Aging

Among circulating markers of inflammaging, interleukin-6 (IL-6) is the most consistently validated across various studies. Elevated IL-6 has been independently associated with all-cause, cardiovascular, and cancer-related mortality in community-dwelling older adults, as demonstrated in the Rancho Bernardo Study [167], PolSenior [168], and Framingham Heart Study Offspring cohorts [169]. A composite inflammaging index incorporating IL-6, IL-10 and CXCL9 has been shown to be more effective than IL-6 alone at predicting long-term mortality among hospitalized older adults when combined with frailty status [170]. Other markers of systemic inflammation, including C-reactive protein (CRP) and tumor necrosis factor-alpha (TNF-α), are also elevated with aging and have been shown to decrease in response to sustained caloric restriction, suggesting their utility as additional indicators of inflammaging trajectory [171]. Age-related remodeling of peripheral T cell subsets, particularly the contraction of the naïve T cell compartment and the expansion of terminally differentiated CD28^−^CD57^+^ and TEMRA CD8^+^ T cells, carries prognostic value independently of chronological age. In the Framingham Heart Study Offspring cohort, a higher CD4/CD8 ratio was associated with lower all-cause mortality, whereas an elevated proportion of CD8^+^CD25^+^FoxP3^+^ regulatory T cells was associated with higher mortality [169]. The accumulation of TEMRA cells has been linked to reduced antibody responses to mRNA-based SARS-CoV-2 vaccination in frail older adults [172]. Furthermore, the proportions of CD31^+^ naïve CD4^+^ and CD8^+^ T cells were found to correlate more strongly with responsiveness to multiple vaccines, including those for influenza, pneumococcus, and SARS-CoV-2, than chronological age itself [173]. Supporting these findings, multi-omic profiling of peripheral blood leukocytes revealed progressive reprogramming of T cells towards a Th2 bias with age. This is mechanistically linked to dysregulated effector B cell responses and altered immunoglobulin subclass production following vaccination [158].

### 5.2. Adapting Vaccine Strategies to the Immunosenescent Host

Due to the reduced magnitude and duration of humoral and cellular responses to standard-dose vaccines in older adults, as outlined in Section 3.4, Section 4.1, and Section 4.2, specific vaccine formulation strategies have been developed to address these limitations. A high-dose inactivated influenza vaccine containing a fourfold higher antigen dose than the standard formulation has been shown to provide substantially improved protection against influenza-related hospitalization in a pooled analysis of two large randomized trials involving adults aged 65 years and over [174]. The adjuvanted recombinant subunit vaccine combining the gE antigen (glycoprotein E of varicella-zoster virus) with the AS01B adjuvant system has been shown to be over 90% more effective in preventing herpes zoster in adults aged 70 years and older, with protection maintained above 89% (i.e., vaccine efficacy compared to placebo of 89.8% in the ZOE-70 trial and 97.2% in the ZOE-50 trial) in the ZOE-50 and ZOE-70 phase 3 trials [175]. An observational study has also suggested that AS01B-adjuvanted vaccination against shingles and respiratory syncytial virus (RSV) may be associated with a reduced risk of dementia, potentially through modulation of neuroinflammation. However, this hypothesis requires confirmation in controlled trials [176]. The finding that proportions of naïve T cells predict multi-vaccine responsiveness more reliably than chronological age suggests that pre-vaccination immunophenotyping could inform personalized vaccination strategies in the future, although there is currently insufficient evidence to support this in clinical practice [173].

### 5.3. Pharmacological Strategies Targeting Immune Aging

#### 5.3.1. mTOR Inhibition

The overactivation of the mTOR complex 1 contributes to cellular senescence and impairs key immune functions, including autophagy and the responsiveness to type I interferons. These are mechanisms that are directly relevant to the dysfunction of dendritic cells and T cells. Clinical trials of low-dose mTOR inhibitors in adults aged 65 years and over have demonstrated improved antibody responses to influenza vaccination, a reduced proportion of PD-1-expressing CD4^+^ and CD8^+^ T cells, and a lower rate of respiratory tract infections [177,178,179]. These results provide proof of concept that selective, low-dose mTOR inhibition can partially restore immune function in older individuals. However, no trial has yet demonstrated improvement in clinical endpoints such as mortality.

#### 5.3.2. Senolytic Therapy

Senescent cells accumulate with age in immune and non-immune tissues and drive chronic inflammation through SASP secretion. The first clinical trial on senolytic agents—a phase 1, open-label, single-arm study combining dasatinib and quercetin performed in nine adults with diabetic kidney disease—demonstrated a significant reduction in the burden of senescent cells in the adipose tissue and epidermis, and in the amount of circulating SASP factors, including IL-1α, IL-6, MMP-9, and MMP-12 within days of treatment [180]. A subsequent randomized placebo-controlled feasibility trial of intermittent dasatinib and quercetin in 12 participants with idiopathic pulmonary fibrosis confirmed the absence of serious adverse events in the senolytic group. However, statistically significant changes in functional endpoints were not detected in this study [181].

A population of p21^+^TREM2^+^ senescent macrophages, which has been characterized in aged mouse livers and identified in human cirrhotic liver tissue, can be selectively cleared by senolytics. This reduces hepatic steatosis and chronic inflammation in preclinical models [146]. However, whether senolytic clearance of immune senescent cells improves vaccine responsiveness or reduces infection risk in older adults has yet to be tested in an adequately powered clinical trial.

#### 5.3.3. Thymic Regeneration

Declining thymopoiesis is a primary driver of reduced naïve T cell output and TCR repertoire diversity. In a pilot clinical trial (the TRIIM study), nine healthy men aged 51–65 years were treated with a combination of recombinant human growth hormone, metformin, and DHEA for 12 months. Thymic regrowth was confirmed using MRI alongside increases in the proportions of naïve T cells and recent thymic emigrants, as well as a reversal of epigenetic aging markers across four independent DNA methylation clocks [182]. These are the first human data to demonstrate that thymic function and epigenetic markers of immune aging can be modified simultaneously by a pharmacological intervention. However, the small, all-male cohort, single-arm design, and use of multiple co-interventions limit the conclusions that can be drawn.

While recombinant IL-7 stimulates peripheral T cell expansion in aged humans, it has not been shown to increase recent thymic emigrant production. This indicates that the restoration of naïve T cell output likely requires structural thymic regeneration rather than peripheral cytokine supplementation [183].

### 5.4. Lifestyle Interventions

Structured physical exercise reduces markers of cellular senescence in circulating immune cells, including p16, p21, cGAS, and TNF-α in CD3^+^ T cells, and also decreases circulating SASP-associated proteins [184]. In older adults, regular moderate-intensity aerobic exercise is associated with higher proportions of naïve T cells and lower proportions of terminally differentiated CD28^−^ memory T cells than in age-matched sedentary individuals. It also increases NK cell cytotoxic activity and improves neutrophil chemotactic responses. These effects are directly relevant to the functional deficits of innate and adaptive immune cells [185].

In the two-year CALERIE-2 randomized trial of sustained moderate (25%) caloric restriction, participants in the intervention group maintained unchanged or had increased thymic volume. They also had higher proportions of circulating recent thymic emigrants than the control group. Suppression of the PLA2G7 gene was identified as a potential immunometabolic mediator of these effects [186]. Caloric restriction reduced circulating C-reactive protein (CRP) and tumor necrosis factor (TNF)-α levels relative to the control group [171] and was associated with a measurable deceleration in biological aging as assessed by the DunedinPACE epigenetic clock [187]. These findings suggest that, when implemented without inducing malnutrition, sustained moderate caloric restriction can positively modify various immune aging endpoints in humans. These results provide clinical corroboration for the mechanistic findings from animal models described in the preceding sections.

Chronic psychosocial stress has been associated with accelerated immunosenescence in humans. This includes lower proportions of naïve CD4^+^ and CD8^+^ T cells, as well as higher proportions of terminally differentiated T cell subsets. This was demonstrated in a large, nationally representative, longitudinal study of adults aged 50 years and over [188]. These associations were partly mediated by neuroendocrine pathways involving the hypothalamic–pituitary–adrenal axis and the sympathetic nervous system. This is consistent with the neuroimmune interactions documented in splenic and thymic innervation in rodent models [189]. Further evidence suggests that the habitual use of adaptive emotion regulation strategies, such as cognitive reappraisal, may mitigate the impact of life stress on immune aging markers. Individuals experiencing high chronic stress who habitually employed reappraisal exhibited lower proportions of late-differentiated CD8^+^ T cells and NK cells, as well as lower circulating IL-6 compared to equally stressed individuals who employed this strategy less frequently [190].

### 5.5. Limitations and Future Directions

Several important constraints limit the clinical translation of the reviewed evidence. Most biomarker studies are cross-sectional, and most therapeutic trials in immune aging are small-scale and short-term. No intervention has yet demonstrated an improvement in hard clinical outcomes in an adequately powered randomized controlled trial. Immune aging is markedly heterogeneous, driven by variation in cytomegalovirus (CMV) status, sex, metabolic status, and socioeconomic exposures. The mechanistic signatures identified in animal models, including lymph node stromal dysfunction, asynchronous organ-level involution, and sex-dependent alterations of T cells, lack systematic parallel characterization in human tissues. Future research priorities should include developing and validating composite immune age indices against clinical outcome, conducting adequately powered trials of senolytic, mTOR-targeting, and thymic restoration interventions with pre-specified immune biomarker endpoints, and rigorously evaluating modifiable lifestyle and psychosocial determinants of immunosenescence in diverse human cohorts.

## 6. Conclusions

In summary, aging of the immune system is a complex process that involves both structural changes in lymphoid organs and functional alterations in immune cells. With time, the bone marrow, thymus, spleen, and lymph nodes undergo gradual involution. This leads to disrupted spatial organization, weaker interactions between stromal cells and lymphocytes, and reduced capacity to produce naïve immune cells. At the same time, important changes occur in both innate and adaptive immune cells. Neutrophils, macrophages, and dendritic cells exhibit reduced efficiency in key functions, including phagocytosis, chemotaxis, antigen presentation, and cytokine production. In parallel, T and B lymphocyte populations shift toward fewer naïve cells and a higher proportion of memory cells. This is associated with reduced receptor diversity, impaired proliferation, and a weaker ability to generate effective antibody responses. An important feature of immune aging is the accumulation of senescent cells. These cells exhibit altered function, resist apoptosis, and secrete SASP, thereby contributing to a persistent, low-grade inflammatory state. These combined changes result in higher susceptibility to infections, reduced responses to vaccination, and increased risk of cancer, autoimmune diseases, and chronic inflammatory conditions.

## Figures and Tables

**Table 1:** Aging-related changes in the immune system: Comparative evidence from human and animal (mouse and rat) studies, and shared molecular mechanisms.

Lymphoid Organ/Cell Type	Human Evidence	Animal Evidence (Mice/Rats)	Shared Mechanisms
**LYMPHOID ORGANS**			
**BONE** **MARROW**	HSCs: ↓ clonogenicity, ↓ self-renewal, myeloid bias, ↓ naïve T cell generation [26,27]; MSCs: enlarged granular morphology, ↑ β-Gal, SASP, ↑ ROS, ↑ p16/p21, ↑ IL-6/IL-8, ↓ IDO activity, ↓ M1 macrophage inhibition [33]; ↑ Adipogenesis [34], ↑ Apoptotic cells [35]; ↑ NK precursors/T precursors ratio, ↓ CD56^bright^ NK cells, ↑ CD56^dim^CD57^+^ NK cells, telomere loss, ↓ telomerase activity [38,39]	MICE: ↑ Adipogenesis with ↑ RANKL expression, Pref-1^+^RANKL^+^ preadipocytes → osteoclastogenesis, myeloid bias [7,36]; ↑ Mac-1^+^ myeloid macrophages with ↓ TNF-α production [37]; ROS from senescent cells → DNA breaks in HSCs, ↓ DNA repair → functional exhaustion [41]	HSC myeloid bias, MSC dysfunction and SASP, ↑ adipogenesis, ↑ senescent cells and ROS, ↓ naïve lymphocyte output
**THYMUS**	Max size ~1 yr after birth, ↓ 3%/yr until middle age [52,52,53,54]; TECs: SA-β-Gal^+^, γH2AX^+^, 8-oxoguanine^+^ → DNA damage and senescence [67]; ↑ SASP/IL-6 → cortical CD4^+^/CD8^+^ thymocyte loss [68]; Thymic involution → ↑ cancer and infectious disease risk [62]; Atrophy reversible with adequate nutrition [59]	MICE: ↓ Naïve T cell production, ↓ Tregs → autoreactive lymphocytes enter circulation [54,63]; Atypical TEC states: perimedullary clusters, epithelial mesenchymal transition, ↓ FOXN1 [64]; Corticomedullary junction atrophy, fibroblast/fat expansion, ↑ perivascular space [65,66]; RATS: ↓ Thymus weight (4–20 months), ↓ thymocytes, ↑ norepinephrine → adrenergic disruption of stromal microenvironment, ↓ T cell differentiation [69]; Global DNA hypomethylation, ↓H3K9 methylation, ↑ genomic instability, ↑ apoptosis, disrupted T lymphocyte composition, ↓ MYC expression [70]	↓Thymopoiesis and naïve T cell output, stromal microenvironment disruption, ↑senescent cells and SASP, ↓TCR repertoire diversity, ↑autoimmunity risk
**SPLEEN**	Elastin fiber shortening, fragmentation, thickening in capsule [75]; Capsule thinning, ↓B-cell follicle number/size in individuals >70 yrs [81]; ↓Marginal zone B cells → poor response to *S. pneumoniae* polysaccharides [82]	MICE: ↓ Cellularity, blurred T/B compartment boundaries, altered marginal zone macrophages [76]; ↓ CCL21, ↓ CXCL13 → impaired CD4^+^ T cell recruitment [18]; ↓ FDC density, ↓ germinal center formation [76,77,78,79,80]; ↑ Treg, ↑ Tex, ↑ Th1, ↑ TEM, ↑ TCM, ↑ memory B cells, ↓ naïve CD4^+^/CD8^+^ T cells, ↓ CD4^+^/CD8^+^ ratio; ↑ p16^INK4a^, ↑ p21^Waf1^/^Cip1^, ↑ ROS in T and B cells [83]; Splenic stromal cells: ↑ IL-6 wi th age [84]; RATS: (21–27 months): ↓ sympathetic noradrenergic innervation (↓ TH+ fibers, ↓ norepinephrine from 17 months), ↓ OX19^+^ T cells, ↓ ED3^+^ macrophages [88]; ↓ T cell proliferative response (PHA), ↓ IL-2, ↓ OX-22/CD45RC on CD4^+^ T cells; macrophage-mediated T cell suppression [87]; NK activity stable until 18 months, then rapid decline, macrophage-derived PGE2 suppresses NK, macrophage removal partially restores cytotoxicity [86]; Old rats (18–24 months): splenic atrophy, ↓ organ weight, ↓ follicle size, ↑ oxidative stress (↓ T-SOD, ↑ MDA), ↓ T/B lymphocytes/macrophages in PALS/marginal zone/follicles, ↑ mast cells [85]	↓ Germinal center formation ↓ naïve T/B cells, ↑ memory cells ↑ senescent cells, ↑ ROS, ↑ CDKI expression, stromal dysfunction → ↑ IL-6, chronic inflammation
**LYMPH** **NODES**	Progressive ↓ B and T lymphocytes, ↑ connective tissue replacing lymphoid tissue [94]; Progressive degeneration in head/neck LNs [95]; ↓ Germinal centers, hyalinization, fibrosis, adipocyte deposition, ↓ HEVs [93]; 5× lower antibody production after influenza vaccination (65–75 vs. 18–36 yrs) [104]; ↓ IgM/IgG production, ↓ antibody affinity [101,102,103]; ↓ FDC antigen capture/retention [99,100] Lipomatosis → HEV remodeling, ↓ lymphatic network [98]	MICE: ↓ Naïve CD4^+^/CD8^+^ T cells, ↓ naïve B cells, ↑ Tex, ↑ Tfh, ↑ Th1, ↑ CTL, ↑ TCM, ↑ TEM, ↑ memory B cells, ↑ p16^INK4a^, ↑ p21^Waf1/Cip1^ in CD45^+^ leukocytes, ↑ ROS in FRCs and LECs [83]; Skin draining LNs atrophy from 6–9 months, deep LNs from 18–20 months, FRC network degradation, naïve T cell emigration (CCR7^lo^S1P1^hi^), impaired vaccination response from 7–8 months [105]; FRCs, LECs, BECs: ↑ mROS, ↓ mitochondrial membrane potential, ↓ naive T cell survival suport, NAC, mitoquinone, urolithin a restore function in vitro and in vivo [106]; 10× fewer germinal center B cells 10 days post-immunization vs. young mice, ↓ DC-associated CD80/CD86 [104]; RATS: (5–37 months): progressive structural disorganization of mesenteric LNs, ↓ cortical cellularity, ↓ germinal centers, medullary sinus distension, ↑ fibroblast infiltration [96]; ↓ Total T cells, CD4^+^/CD8^+^ shift toward CD8^+^, naïve→memory/activated shift, ↑ Tregs, males more affected, ↑ CD28^−^CD11b^+^ senescent cells, ↑ PD-1^high^ exhausted CD8^+^ T cells, correlates with peripheral blood [107]	↓ Germinal center formation, ↓ naïve T/B cells, ↑ memory/exhausted cells, stromal dysfunction (FRC, LEC, FDC), ↑ senescent cells, ↑ ROS, ↓ vaccine induced humoral immunity
**INNATE IMMUNE CELLS**			
**NK CELLS**	Peripheral blood: 48% depletion of CD56^bright^ subset → ↓ regulatory cytokines/chemokines [109]; ↑ CD56^dim^CD57^+^ cells: high cytolytic capacity but ↓ IL-12/IL-18 responsiveness [110]; ↓ NKp30 expression → ↓ cytotoxic function, ↓ DC signaling [111]; Telomere shortening → ↓ proliferative potential [38,39]	MICE: Multi-organ single-cell cytometry (12 tissues): ↓ NK cell numbers in peripheral organs incl. spleen and LNs [113]; Impaired NK maturation in BM due to defective stromal signals, IL-15/IL-15Rα → numeric expansion but cells remain immature, no protection against mousepox [112];	↓ NK cytotoxicity, telomere shortening, shift toward mature/differentiated subsets
**DENDRITIC CELLS**	PDCs (65–90 yrs): ↓ IFN-I and IFN-III secretion, ↓ IRF-7 phosphorylation, ↓ perforin/granzyme induction in CD8^+^ T cells [116]; MDCs: ↓ phagocytosis, ↓ migration (MIP-3β, SDF-1), ↑ TNF-α/IL-6 (LPS), ↓ PI3K pathway [117]; ↑ Reactivity to self-antigen (human DNA) → chronic inflammation [118]; ↓ Langerhans cells in epidermis, ↓ TNF-α-induced migration [120];	MICE: ↓ FcγRII on FDCs → ↓ immune complex retention → ↓ B cell proliferation [99,100,119]; ↓ LC numbers/density, impaired maturation, ↓ induction of T cell proliferation, altered miRNA profile (TGF-β signaling) [121]; scRNA-seq of LNs after vaccination: DC migration defect (CCR7), oral delivery of yeast-derived β-glucan-containing nanoparticles: ↑CCR7 → ↑ LN trafficking → ↑ vaccine immunity [122]; ↓ Cross presentation, ↓ CD8^+^ T cell priming, mitochondrial dysfunction (↑ ROS, ↓ membrane potential), ROS reduction partially restores cross-presentation [123]; Aged pDCs: ↓ IFN-α → defective HSV-2 clearance, ↓ IRF-7 upregulation during TLR9 activation, antioxidant treatment partially restores IFN-α [125]; ↓ Tfh differentiation after vaccination due to poor cDC2 activation, TLR7 agonist restores cDC2 and germinal centers [104]; Aged DCs against melanoma: ↓ T cell stimulation, ↓ CCR7-CCL21 migration in vivo, ↓ DC-SIGN [124]	↓ Antigen capture and presentation, ↓ Migration (CCR7-CCL21 axis), ↓ IFN-I/IRF-7 signaling in pDCs, mitochondrial dysfunction, ↑ROS, cDC2 dysfunction → ↓ Tfh differentiation (both species)
**NEUTROPHILS**	↓ Phagocytosis [127]; abnormal adhesion and chemotaxis [128]; ↓ NET release [129]; TLR dysfunction [130]; GM-CSF/(Jak/STAT): extends lifespan in young but not in elderly individuals [131]; Age-related changes in neutrophil function → ↑ morbidity and mortality [132]; Epigenetic changes → functional abnormalities [127]	MICE: ↓ Phagocytosis of opsonized *S. pneumoniae*, ↓ intracellular killing (↓ CRAMP), ↓ chemotaxis, ↓ NETosis, ↓ wound healing (↓ ICAM-1), neutrophil intrinsic defects confirmed by adoptive transfer, ↓ TLR4/LPS-priming pathway (↓ PIP3, ↓ MyD88) [133]	↓ Phagocytosis, ↓ chemotaxis, ↓ NET release, TLR dysfunction, epigenetic alterations; ↓ Intracellular bacterial killing; ↓ wound healing (↓ ICAM-1), species-specific signaling: ↓ TLR4/LPS → ↓ PIP3/↓ MyD88 (mice) vs. ↓ GM-CSF/ (Jak/STAT) (humans)
**MACROPHAGES/MONOCYTES**	↓ Phagocytosis, ↓ migration, ↓ chemotaxis in MDMs from donors >50 yrs, ↓ c-MYC, ↓ USF-1 [142]; Human monocytes: ↓ IL-6, ↓ TNF-α after TLR1/TLR2 stimulation [136,137]; ↓ HLA class II on monocytes [139]; Senescent p21^high^ macrophages present in human cirrhotic liver [146]; ↓ CD206 (M2), ↑ iNOS (M1 shift), SASP → paracrine M1 polarization, ↓ IL-4/IL-13/STAT6 → ↓M2 [143,144];	MICE: Aged mice: ↓ TLR4 mediated cytokine production [138]; ↓ MHC class II [140]; ↓ c-MYC and ↓USF-1 (shared with humans) [142]; Senescent p21^high^ macrophages accumulate in livers of aged mice [146]; In vitro peritoneal macrophage model (7–14 days): ↑p16^INK4A^, ↑ p21^CIP1^, ↑ SASP, ↓ phagocytosis, ↑ glycolysis, CB3 peptide prevents p21^CIP1^ upregulation [147]; ↓ MerTK signaling, ↓ autophagy → impaired efferocytosis → DAMPs → ↑ inflammation [145]	↓ Phagocytosis, ↓ MHC/HLA class II, M1 polarization shift, ↓ c-MYC/USF-1 axis (humans and mice), senescent p21^high^ macrophage accumulation (humans and mice), SASP secretion
**ADAPTIVE IMMUNE CELLS**			
**T LYMPHOCYTES**	↓ Naïve T cells, ↑ memory T cells, ↑ senescent cells [6]; CD28^−^CD57^+^ T cells accumulate after repeated antigen stimulation [149]; TEMRA CD8^+^ T cells: CD57^+^/KLRG1^+^, γH2AX^+^, SASP, mitochondrial dysfunction, ↓ ATP (p38 MAPK → ↓ autophagy), ↑ TNF-α/IFN-γ, linked to CMV [150,151,152]; SA-β-Gal^+^ CD8^+^ T cells: 64% in 60 yr olds, dysfunctional telomeres, p16 senescence, ↓proliferation [157]; Supercentenarians: CD4^+^ T cells acquire cytotoxic phenotype [153]; Naïve T cells show greatest transcriptomic age changes (RNA-seq), CD4^+^/CD8^+^ reprogramming → Th2 bias [158]	MICE: Mathematical modeling: naïve T cells maintained by ↑lifespan, not proliferation as thymus involutes, CD4^+^ divide rarely, young CD8^+^ recruited early to memory pool [154]; Splenic CD4^+^ T cells: drastic ↓TCR diversity (clonal expansions without antigen), bone marrow preserves diversity [155]; TCRδ^CreER^ mice: ↓organelle function in naïve T cells, ↑PD-1, ↑LAG-3, ↑TIM-3 in memory cells, CD4^+^ memory proapoptotic vs. CD8^+^ memory antiapoptotic [156]	↓Naïve T cells, ↑memory/exhausted T cells, ↓TCR diversity, telomere shortening, mitochondrial dysfunction, ↑PD-1, ↑LAG-3, ↑TIM-3 (mice and humans), asymmetric apoptotic regulation CD4^+^ vs. CD8^+^ (mice and humans)
**B LYMPHOCYTES**	↓ Naïve B cells, ↑ memory B cells [101]; ↓ BCR diversity (86–94 yr individuals) correlated with frailty, lower survival, vitamin B12 deficiency [101]; ↓ Specific antibody production, ↑ nonspecific antibodies [101]; B-1 cells (>65 yrs): ↓ XBP-1, ↓ BLIMP-1, ↑ PAX-5 → ↓ plasma cell differentiation [166]; Effector memory CD27^−^ B cells after influenza vaccine: ↓ ROS signaling, altered IGHG (↑ IgG2/↓ IgG3) → ↓ early antiviral response [158]	MICE: ↓ B cell proliferation with BM stromal cells after 24 months; ↓ stromal cell support; ↓ IL-7 production by stromal cells [160,161,162]; B lymphocyte precursors: ↓ expansion in response to IL-7 [163]; ↑ Memory B cells in spleen and LNs of aged mice [83]	↓ Naïve B cells, ↑ memory B cells, ↓ BCR diversity, ↓ germinal center B cell responses, ↓ antibody production and affinity

ATP: adenosine triphosphate; BCR: B cell receptor; BEC: blood endothelial cell; BLIMP-1: B lymphocyte-induced maturation protein 1; BM: bone marrow; CB3: thioredoxin-1 mimetic anti-inflammatory peptide; CCL21: C-C motif chemokine ligand 21; CCR7: C-C chemokine receptor type 7; cDC2: conventional type 2 dendritic cell; CDKI: cyclin-dependent kinase inhibitor; CMV: cytomegalovirus; CRAMP: cathelicidin-related antimicrobial peptide; CTL: cytotoxic T lymphocyte; CXCL13: C-X-C motif chemokine ligand 13; DAMP: damage-associated molecular pattern; DC: dendritic cell; DC-SIGN: DC-specific ICAM-3-grabbing non-integrin; FDC: follicular dendritic cell; FOXN1: forkhead box protein N1; FRC: fibroblastic reticular cell; γH2AX: phosphorylated histone H2AX (DNA damage marker); GM-CSF: granulocyte macrophage colony-stimulating factor; HEV: high endothelial venule; HLA: human leukocyte antigen; HSC: hematopoietic stem cell; HSV-2: herpes simplex virus type 2; ICAM-1: intercellular adhesion molecule 1; IDO: indoleamine 2,3-dioxygenase; IFN: interferon; IGHG: immunoglobulin heavy chain gamma; IL: interleukin; iNOS: inducible nitric oxide synthase; IRF-7: interferon regulatory factor 7; Jak/STAT: Janus kinase/signal transducer and activator of transcription; KLRG1: killer cell lectin-like receptor G1; LAG-3: lymphocyte activation gene 3; LC: Langerhans cell; LEC: lymphatic endothelial cell; LN: lymph node; LPS: lipopolysaccharide; MAPK: mitogen-activated protein kinase; MDA: malondialdehyde; MDC: myeloid dendritic cell; MDM: monocyte-derived macrophage; MerTK: Mer tyrosine kinase; MHC: major histocompatibility complex; MIP-3β: macrophage inflammatory protein-3 beta; mROS: mitochondrial reactive oxygen species; MSC: mesenchymal stromal cell; MYC: MYC proto-oncogene transcription factor; MyD88: myeloid differentiation primary response 88; NAC: N-acetylcysteine; NET: neutrophil extracellular trap; NETosis: process of NET formation and release; NK: natural killer; NKp30: natural killer cell p30-related protein; PALS: periarteriolar lymphoid sheath; PAX5: paired box protein 5; pDC: plasmacytoid dendritic cell; PD-1: programmed cell death protein 1; PGE2: prostaglandin E2; PHA: phytohemagglutinin; PI3K: phosphoinositide 3-kinase; PIP3: phosphatidylinositol (3,4,5)-trisphosphate; Pref-1: preadipocyte factor 1; RANKL: receptor activator of NF-κB ligand; ROS: reactive oxygen species; S1P1: sphingosine-1-phosphate receptor 1; SA-β-Gal: senescence-associated βgalactosidase; SASP: senescence-associated secretory phenotype; SDF-1: stromal cell derived factor 1; STAT6: signal transducer and activator of transcription 6; TCM: central memory T cell; TCR: T cell receptor; TEC: thymic epithelial cell; TEM: effector memory T cell; TEMRA: terminally differentiated effector memory T cell re-expressing CD45RA; Tex: exhausted T cell; Tfh: follicular helper T cell; TGF-β: transforming growth factor beta; TH: tyrosine hydroxylase; Th1: T helper 1 cell; TIM-3: T cell immunoglobulin and mucin-domain containing-3; TLR: toll-like receptor; TLR4: toll-like receptor 4; TNF-α: tumor necrosis factor alpha; Treg: regulatory T cell; T-SOD: total superoxide dismutase; USF-1: upstream transcription factor 1; XBP-1: X-box binding protein 1. Up arrow for: an increase, down arrow for: a decrease, and side arrow for: leads to.

## Data Availability

No new data were created or analyzed in this study. Data sharing is not applicable to this article.
